# miR-504 mediated down-regulation of nuclear respiratory factor 1 leads to radio-resistance in nasopharyngeal carcinoma

**DOI:** 10.18632/oncotarget.4138

**Published:** 2015-05-14

**Authors:** Luqing Zhao, Min Tang, Zheyu Hu, Bin Yan, Weiwei Pi, Zhi Li, Jing Zhang, Liqin Zhang, Wuzhong Jiang, Guo Li, Yuanzheng Qiu, Fang Hu, Feng Liu, Jingchen Lu, Xue Chen, Lanbo Xiao, Zhijie Xu, Yongguang Tao, Lifang Yang, Ann M. Bode, Zigang Dong, Jian Zhou, Jia Fan, Lunquan Sun, Ya Cao

**Affiliations:** ^1^ Cancer Research Institute, Xiangya School of Medicine, Central South University, Changsha, China; ^2^ Key Laboratory of Carcinogenesis and Invasion, Ministry of Education, Changsha, China; ^3^ Key Laboratory of Carcinogenesis, Ministry of Health, Changsha, China; ^4^ Molecular Imaging Research Center, Xiangya Hospital, Central South University, Changsha, China; ^5^ Center for Molecular Medicine, Xiangya Hospital, Central South University, Changsha, China; ^6^ Department of Oncology, Xiangya Hospital, Central South University, Changsha, China; ^7^ Department of Otolaryngology Head and Neck Surgery, Xiangya Hospital, Central South University, Changsha, China; ^8^ Department of Radiology, Xiangya Hospital, Central South University, Changsha, China; ^9^ Metabolism Endocrinology Research Institute, The Second Xiangya Hospital, Central South University, Changsha, China; ^10^ The Hormel Institute, University of Minnesota, Austin, MN, USA; ^11^ Department of Live Surgery, Liver Cancer Institute, Zhongshan Hospital, Fudan University, Shanghai, China

**Keywords:** miR-504, radio-resistance, nuclear respiratory factor 1 (NRF1), biomarker, nasopharyngeal carcinoma (NPC)

## Abstract

microRNAs (miRNAs) are involved in the various processes of DNA damage repair and play crucial roles in regulating response of tumors to radiation therapy. Here, we used nasopharyngeal carcinoma (NPC) radio-resistant cell lines as models and found that the expression of miR-504 was significantly up-regulated. In contrast, the expression of nuclear respiratory factor 1 (NRF1) and other mitochondrial metabolism factors, including mitochondrial transcription factor A (TFAM) and oxidative phosphorylation (OXPHOS) complex III were down-regulated in these cell lines. At the same time, the Seahorse cell mitochondrial stress test results indicated that the mitochondrial respiratory capacity was impaired in NPC radio-resistant cell lines and in a miR-504 over-expressing cell line. We also conducted dual luciferase reporter assays and verified that miR-504 could directly target NRF1. Additionally, miR-504 could down-regulate the expression of TFAM and OXPHOS complexes I, III, and IV and impaired the mitochondrial respiratory function of NPC cells. Furthermore, serum from NPC patients showed that miR-504 was up-regulated during different weeks of radiotherapy and correlated with tumor, lymph nodes and metastasis (TNM) stages and total tumor volume. The radio-therapeutic effect at three months after radiotherapy was evaluated. Results indicated that patients with high expression of miR-504 exhibited a relatively lower therapeutic effect ratio of complete response (CR), but a higher ratio of partial response (PR), compared to patients with low expression of miR-504. Taken together, these results demonstrated that miR-504 affected the radio-resistance of NPC by down-regulating the expression of NRF1 and disturbing mitochondrial respiratory function. Thus, miR-504 might become a promising biomarker of NPC radio-resistance and targeting miR-504 might improve tumor radiation response.

## INTRODUCTION

Nasopharyngeal carcinoma (NPC) is a head and neck epithelial malignancy that occurs frequently in Southern China [1]. Radiotherapy is the primary treatment against this carcinoma and radio-resistance, which occurs in NPC patients undergoing radiotherapy, could be predicted by several radiation-resistant biomarkers [2]. Exploring the functions and molecular mechanisms of these biomarkers will help us enhance the radio-therapeutic effects against NPC.

MicroRNAs (miRNAs) comprise a class of short non-coding RNAs, which consist of about 22s nucleotides. Through “seed sequences” (7-8 nucleotides), miRNAs bind to the 3′-untranslated region (3′-UTR) of target mRNA and inhibit or block the expression of target genes at the post-transcriptional level [3, 4]. MiRNAs play a pivotal role in modulating tumor radio-sensitivity or radio-resistance by affecting DNA damage repair, cell cycle checkpoint, apoptosis, radio-related signal transduction pathways and tumor microenvironment [5, 6]. MiR-24, miR-101, miR-205, miR-100, and miR-7 have been reported to be closely associated with tumor radio-resistance [7-11].

MiR-504 has been rarely studied in the cancer research field and most of its functions and roles in carcinogenesis are unknown. Recently it has been reported that tumor suppressor gene *Trefoil factor 1 (TFF1)* can down-regulate the expression of miR-504, which is a negative regulator of p53, so as to activate p53 functions in gastric cancer [12]. Computational analyses for potential targets of miR-504 indicated that this miRNA could imperfectly interact with mRNAs of *FOXP1, TP53INP1, TSC1* and *nuclear respiratory factor 1 (NRF1)* genes. NRF1 is a DNA-binding transcription factor that activates genes which are involved in mitochondrial biogenesis and other fundamental cellular functions, such as protein translation/turnover, DNA synthesis/repair, and cell proliferation [13]. NRF1 increases mitochondrial respiratory capacity and induces expression of a subset of genes governing mitochondrial activity in a cell-type specific manner. NRF1-independent mitochondrial gene expression pathways are regulated by peroxisome proliferator activated receptors, Sp1, and other factors [14].

The *mitochondrial transcription factor A (TFAM)* is the major downstream target gene of NRF1. NRF1 is the transcription factor for all the mitochondrial encoded genes that are required for mitochondrial DNA (mtDNA) transcription and replication [15]. Mitochondria are essential organelles that perform diverse cellular functions, including respiration through oxidative phosphorylation (OXPHOS), which proceeds through the coordinated action of five inner mitochondrial membrane protein complexes [16]. During OXPHOS, sequential oxidation-reduction reactions at complexes I (NADH dehydrogenase), II (succinate dehydrogenase), III (coenzyme Q: cytochrome c-oxidoreductase), and IV (cytochrome c oxidodase) are coupled to the translocation of protons across the inner mitochondrial membrane. The resulting electrochemical gradient is ultimately utilized by complex V (ATP synthase) for the generation of ATP from ADP and inorganic phosphate [17]. TFAM could further influence the functions of five inner mitochondrial OXPHOS complexes (I-V) and modulate mitochondrial metabolism to satisfy diverse cellular energy needs [18].

We used miRNA screening of NPC radio-resistant cell lines and adopted dual luciferase reporter assays to identify the dominant miRNA in radio-resistant cells, validate its target gene(s), and further confirm their role in NPC radio-resistance. Here, we found that miR-504 was significantly up-regulated in NPC radio-resistant cell lines. We explored the possible mechanisms of miR-504 in down-regulating the expression of NRF1 and its downstream TFAM and OXPHOS complexes, disturbing the functions of mitochondrial respiratory chain, and influencing the radio-resistant characteristics of NPC cells. The data suggested that miR-504, which is involved in the regulation of mitochondrial function, might be a novel potential biomarker to predict the response of NPC patients to radiation.

## RESULTS

### Validation of the up-regulation of miR-504 in NPC radio-resistant cell lines

**Figure 1:**
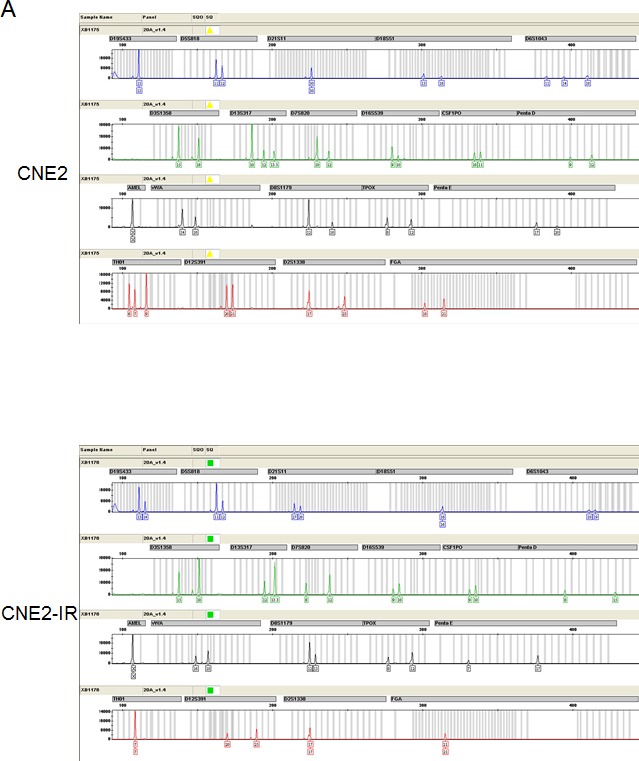

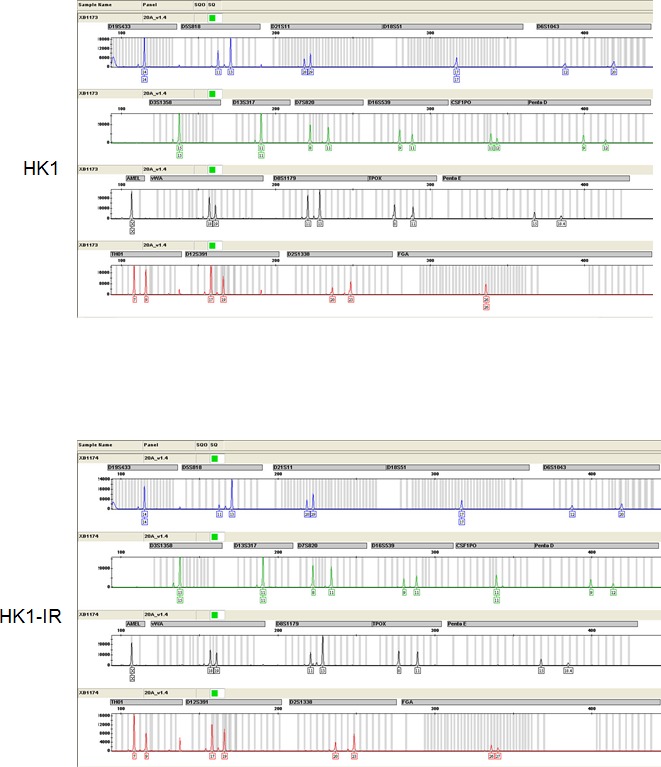

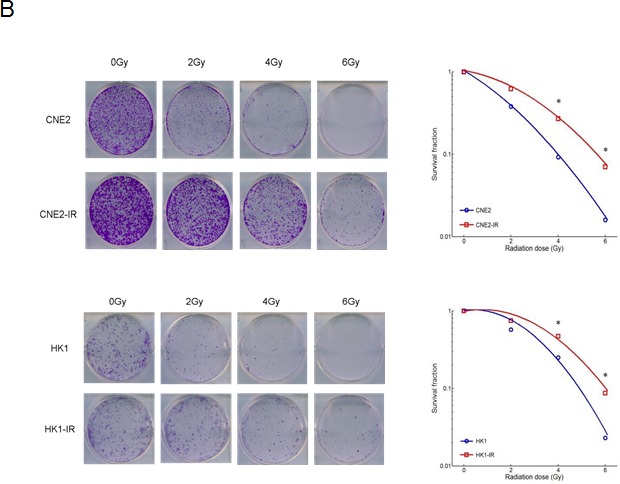

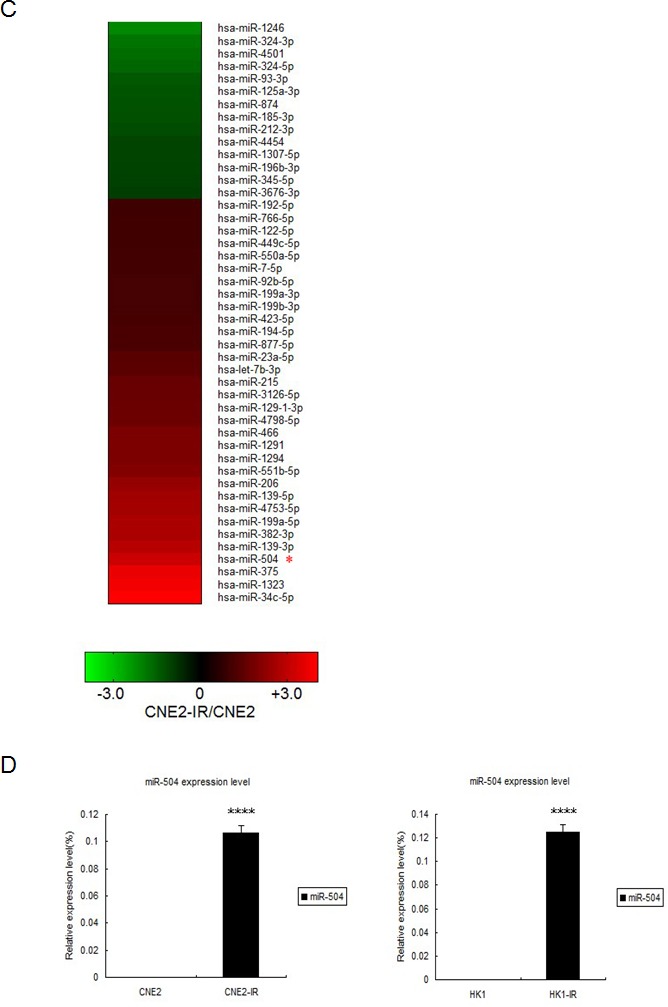

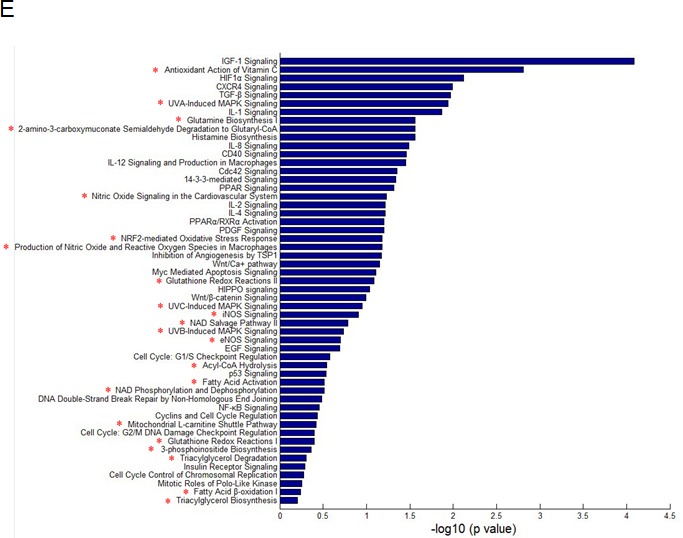
Identification of radio-resistant phenotypes in NPC radio-resistant cell lines and validation of up-regulation of miR-504 in radio-resistant cell lines (A) Cell line short tandem repeat (STR) genotyping of 20 gene loci for CNE2, CNE2-IR, HK1, and HK1-IR cell lines. (B) Results of colony formation assays and survival curves for CNE2, CNE2-IR, HK1, and HK1-IR cell lines. The asterisk (*) indicates a significant difference (*p* < 0.05). (C) A heat map showing the most significantly changed miRNAs in CNE2-IR cells compared with CNE2 cells. Red indicates up-regulated miRNAs and green indicates down-regulated miRNAs. (D) RT-PCR was used to examine the miR-504 expression level in CNE2, CNE2-IR, HK1, and HK1-IR cell lines. U6 snRNA was used as an internal control. The relative expression level of miR-504 was determined as 2^−ΔΔCt^ compared to the Ct value of U6 snRNA. The asterisks (****) indicate a significant difference (*p* < 0.0001). (E) The Ingenuity Pathway Analysis (IPA) software was used to examine the functions of up-regulated miRNAs in CNE2-IR cells. Pathways associated with mitochondrial metabolism are marked with a red asterisk (*).

Using two NPC radio-resistant cell lines, we conducted cell line STR genotyping of 20 gene loci to confirm authenticity and genomic differences. Their gene markers and alleles are shown (Figure 1A, Table S1). In order to identify and verify their radio-resistant phenotypes, we irradiated them with different doses of IR (0, 2, 4, 6 Gy) and examined their survival fractions (SF) by colony formation assay (Figure 1B). The data showed that SFs of the NPC radio-resistant cell lines were much higher than SFs of NPC cell lines after IR treatment. This finding is representative of the radio-resistant phenotype of these radio-resistant cell lines. Next, we used the high-throughput Illumina HiSeq 2000 system to perform miRNA screening of NPC radio-resistant cell lines and selected the most significantly altered miRNAs that were indicated in the heat map (Figure 1C). Next, we used RT-PCR to examine the expression levels of the top five up-regulated miRNAs in two pairs of NPC radio-resistant cell lines (CNE2 and CNE2-IR cells; HK1 and HK1-IR cells). We found that miR-504 was significantly up-regulated in both NPC radio-resistant cell lines (*p* < 0.0001) compared to parent cell lines (Figure 1D). Using several miRNA target prediction databases and Ingenuity Pathway Analysis (IPA) software, we found that the functions of the up-regulated miRNAs are mostly involved in the regulation of mitochondrial metabolism and oxidative and redox response (Figure 1E). This suggests that radio-resistance might have a close relationship with several mitochondrial functions.

### miR-504 promotes cell growth and leads to radio-resistance of NPC cell lines

**Figure 2:**
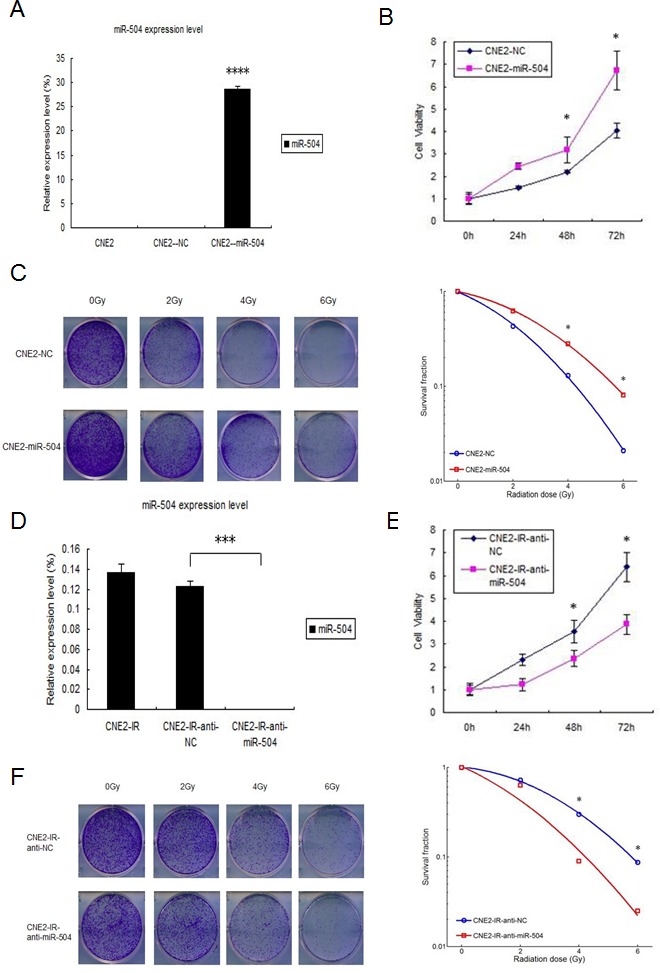

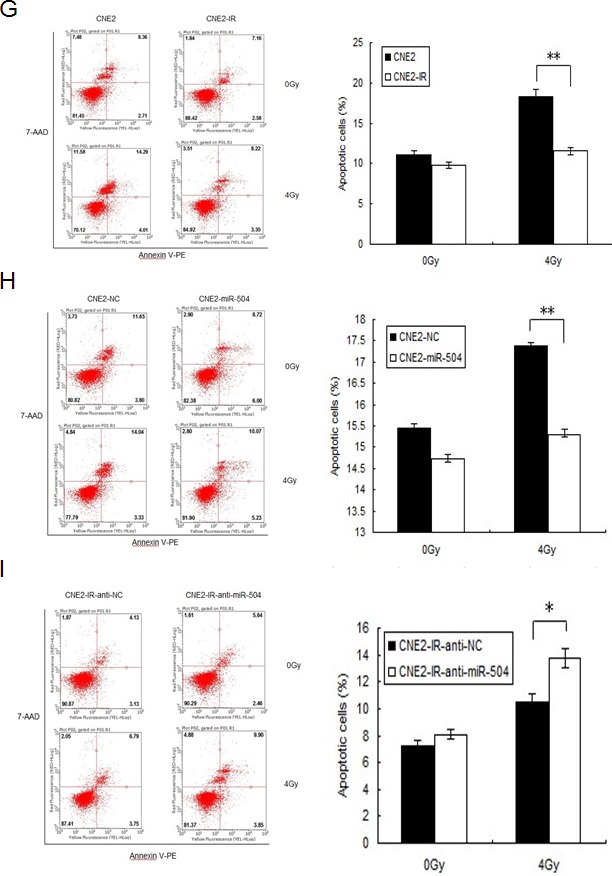
miR-504 promotes NPC cell growth, reduces the number of cells undergoing apoptosis induced by radiation and leads to radio-resistance of NPC cell lines **A.** The transfection efficiency of CNE2 cells transfected with a miR-504 precursor (CNE2-miRNA-504) and a negative control (CNE2-NC) at 48 h after transfection. The asterisks (****) indicate a significant increase (*p* < 0.0001) in miR-504 levels. **B.** MTS assay results to assess and compare viability of CNE2-NC and CNE2-miR-504 cells. Data are shown as mean values ± S.D. of three experiments. The asterisk (*) indicates a significant increase (*p* < 0.05) in viability of CNE2-miR-504 cells. **C.** Results of a colony formation assay and survival curves for CNE2-NC and CNE2-miR-504 cells treated with increasing doses of radiation. The asterisk (*) indicates a significant difference (*p* < 0.05). **D.** The transfection efficiency of CNE2-IR cells transfected with an inhibitor of miR-504 (CNE2-IR-anti-miR-504) and a negative control (CNE2-IR-anti-NC) at 48 h after transfection. The asterisks (***) indicate a significant decrease (*p* < 0.001) in miR-504 expression. **E.** MTS assay results to assess and compare viability of CNE2-IR-anti-NC and CNE2-IR-anti-miR-504 cells. Data are shown as mean values ± S.D. of three experiments. The asterisk (*) indicates a significant decrease (*p* < 0.05) in viability of CNE2-IR-anti-miR-504 cells. **F.** Results of a colony formation assay and survival curves for CNE2-IR-anti-NC and CNE2-IR-anti-miR-504 cells treated with increasing doses of radiation. The asterisk (*) indicates a significant difference (*p* < 0.05). **G.** Assessment of apoptosis of CNE2 and CNE2-IR cells treated with 0 or 4 Gy using Annexin V-PE/7-AAD staining. The asterisks (**) indicate a significant resistance of CNE2-IR cells (*p* < 0.01). **H.** Assessment of apoptosis of CNE2-NC and CNE2-miR-504 cells treated with 0 or 4 Gy using Annexin V-PE/7-AAD staining. The asterisks (**) indicate a significant resistance of CNE2-miR-504 cells (*p* < 0.01). **I.** Assessment of apoptosis of CNE2-IR-anti-NC and CNE2-IR-anti-miR-504 cells treated with 0 or 4 Gy using Annexin V-PE/7-AAD staining. The asterisk (*) indicates an increased sensitivity of CNE2-IR-anti-miR-504 cells to apoptosis (*p* < 0.05).

To explore the biological functions of miR-504, we first transiently transfected a miR-504 precursor and its negative control (NC) into the CNE2 cell line. At 48 h after transfection, we examined the transfection efficiency of miR-504 by RT-PCR (Figure 2A). The miR-504 transfection efficiency was high and was statistically significant (*p* < 0.0001). We next used the MTS assay to examine the effect of miR-504 on cell viability at different time points (0, 24, 48, or 72 h) (Figure 2B). Results showed that miR-504 promoted NPC cell viability in a time dependent manner, especially at the 72 h time point. Also we used the BrdU staining kit to further validate that miR-504 can promote NPC cells growth and proliferation (Figure S1). Moreover, we irradiated CNE2-NC and CNE2-miR-504 cells with different doses of IR (0, 2, 4, or 6 Gy) and examined their SFs by a colony formation assay (Figure 2C). The data showed that the SF of CNE2-miR-504 cells was nearly 3.7-fold higher than that of CNE2-NC cells following 6 Gy treatment, which indicated that miR-504 can increase the radio-resistant phenotype of NPC cells.

To further confirm the role of miR-504 in NPC radio-resistance, we transiently transfected an inhibitor of miR-504 (anti-miR-504) and its negative control (anti-NC) into the CNE2-IR cell line. At 48 h after transfection, we examined the transfection efficiency by RT-PCR (Figure 2D). Compared with CNE2-IR-anti-NC cells, the miR-504 expression level in CNE2-IR-anti-miR-504 cells was significantly down-regulated (*p* < 0.001). We then performed the MTS assay on cells harvested at different time points (0, 24, 48, or 72 h) to examine the effect of anti-miR-504 on cell viability (Figure 2E). Results showed that down-regulating the expression of miR-504 decreased NPC radio-resistant cell viability over time. Moreover, we irradiated CNE2-IR-anti-NC and CNE2-IR-anti-miR-504 cells with different doses of IR (0, 2, 4, or 6 Gy) and examined their SFs by colony formation assay (Figure 2F). The data showed that the SF of CNE2-IR-anti-NC cells was 2.6-fold higher than that of CNE2-IR-anti-miR-504 following 6 Gy treatment, which indicated that inhibiting miR-504 can reverse the radio-resistant phenotype in NPC radio-resistant cells. In order to further support these findings obtained from the CNE2 and CNE2-IR cell models, we repeated the above experiments using HK1 and HK1-IR cells and the results were similar (Figure S2).

### miR-504 reduces radiation-induced apoptosis and allows NPC cells to gain a radio-resistant phenotype

We first exposed CNE2 and CNE2-IR cells to 0 or 4 Gy of IR and then stained them with Annexin V-PE and 7-AAD, and measured apoptosis by flow cytometry (Figure 2G). The data indicated after exposure to 4 Gy IR, CNE2-IR cells exhibited 1.6-fold less apoptosis compared to CNE2 cells (*p* < 0.01), which suggested that the NPC radio-resistant cell line is less sensitive to radiation-induced apoptosis.

To determine whether miR-504 contributes to radio-resistance in these cells, we transiently transfected a miR-504 precursor or its negative control (NC) into CNE2 cells. At 48 h after transfection, cells were exposed to 0 or 4 Gy of IR and then stained with Annexin V-PE and 7-AAD, and apoptosis was measured by flow cytometry (Figure 2H). Results indicated that the cells transfected with miR-504 exhibited 1.1-fold less apoptosis than the negative control-transfected cells (*p* < 0.01) after treatment with 4 Gy IR. These data suggested that miR-504 can facilitate the ability of NPC cells to evade apoptosis induced by radiation. To further validate the role of miR-504, we transfected cells with an inhibitor of miR-504 (anti-miR-504) and assessed radiation-induced apoptosis by flow cytometry (Figure 2I). These results demonstrated that cells expressing negative control (CNE2-IR-anti-NC) exhibited 1.3-fold less apoptosis compared to cells expressing the inhibitor (CNE2-IR-anti-miR-504) after treatment with 4 Gy IR (*p* < 0.05). This suggested that inhibiting miR-504 could increase the sensitivity of radio-resistant NPC cells to apoptosis induced by radiation.

### The serum miR-504 expression levels of NPC patients are elevated during radiotherapy and correlate with TNM stage and total tumor volume

**Figure 3:**
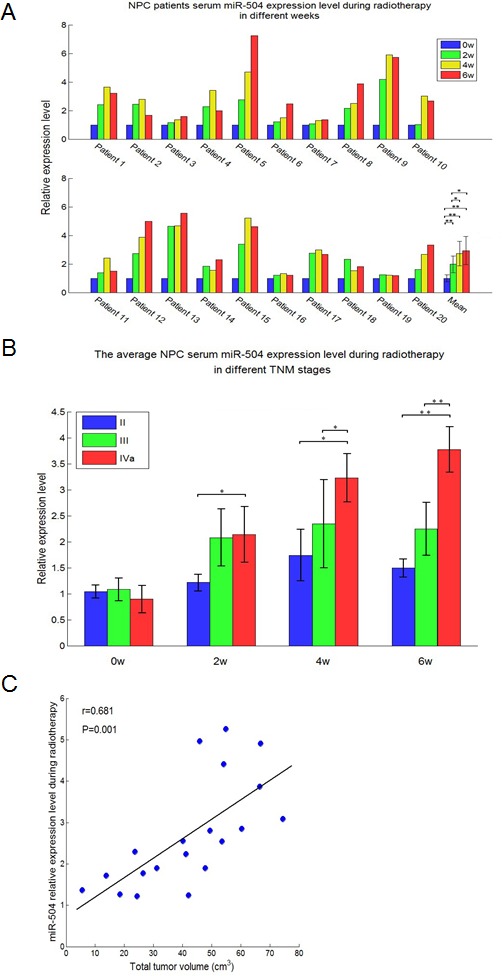

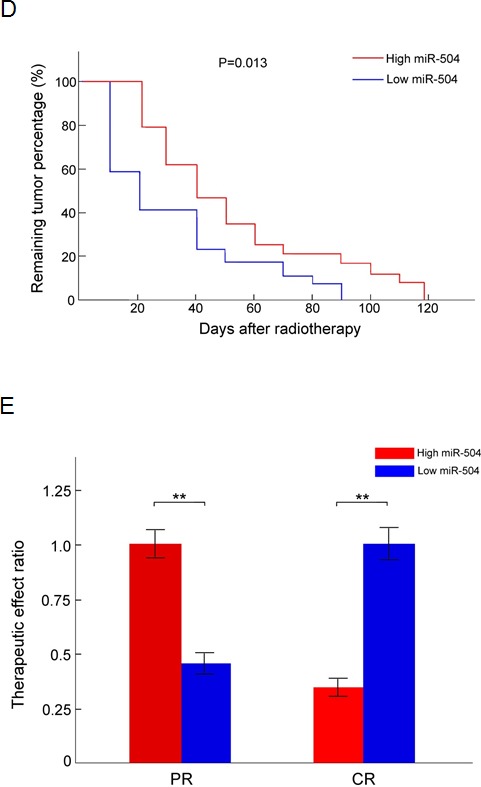
miR-504 expression levels are elevated in the serum of NPC patients at different weeks of radiotherapy and correlate with TNM stages and total tumor volume **A.** Relative expression level of serum miR-504 in 20 NPC patients during radiotherapy at four different weeks (0, 2, 4, 6 weeks). Data are summarized as mean values ± S.D. of three experiments. The asterisk(s) (*, **) indicate a significant increase (*p* < 0.05, *p* < 0.01, respectively) in miR-504 levels. **B.** Average serum miR-504 expression level in NPC patients undergoing radiotherapy at different TNM stages (II, III, IVa). Data are shown as mean values ± S.D. of three experiments. The asterisk(s) (*, **) indicate a significant difference in average miR-504 levels as indicated at different stages (*p* < 0.05, *p* < 0.01, respectively). **C.** Total tumor volume correlates significantly with the average expression level of serum miR-504 in patients undergoing radiotherapy. **D.** The percentage of tumor remaining at different days after radiotherapy in patients who had high or low miR-504 expression levels. **E.** The therapeutic effect ratio of miR-504 high or low expression patients at three months after radiotherapy. PR: partial response; CR: complete response. The asterisks (**) indicate a significantly greater response (*p* < 0.01).

We collected serum samples from 20 Xiangya Hospital NPC patients (Central South University, Changsha, China). For each patient, serum samples were collected at four different time points during radiotherapy (0, 2, 4, 6 weeks) for a total of 80 serum samples from NPC patients in this study. The demographic and clinical features of the NPC patients were obtained (Table S2). We then extracted serum miRNA and used TaqMan^®^ MicroRNA Assays to determine the miR-504 expression level at 0, 2, 4 and 6 weeks during radiotherapy (Figure 3A). Results indicated that the serum miR-504 expression level was elevated in each patient after radiotherapy, but reached its highest peak at different weeks depending on the patient. The average expression level of serum miR-504 for all 20 patients increased time-dependently with radiotherapy and correlated positively with the number of weeks that treatment was administered (Figure 3A). Statistically significant differences were observed at 2, 4 or 6 weeks (*p* < 0.01) compared with week 0.

Next, NPC patients were divided into different groups based on their TNM stage and miR-504 expression level was measured at 0, 2, 4 or 6 weeks during radiotherapy (Figure 3B). Data indicated that the miR-504 expression level in IVa stage patients was higher than levels at stages II or III with statistical significance observed at 6 (*p* < 0.01), 4 (*p* < 0.05) and 2 (*p* < 0.05) weeks compared with week 0. These results suggested that more severe or higher TNM stages of NPC correspond with greater expression of miR-504 induced during radiotherapy to maintain a radio-resistant phenotype. We also analyzed the average expression level of miR-504 at four different times during radiotherapy to examine its relationship with total tumor volume (Figure 3C). The data demonstrated that the average expression level of miR-504 during radiotherapy was positively and significantly correlated (Pearson's correlation coefficient = 0.681; *p* = 0.001) with total tumor volume in NPC patients. These data provide additional evidence that higher expression of miR-504 contributes to radio-resistance of NPC patients.

After the whole process of radiotherapy, we measured the percentage of tumor remaining in patients expressing high levels of miR-504 (9/20 cases, miR-504 expression level elevated more than 3.5-fold after radiotherapy) and in patients expressing low levels of miR-504 (11/20 cases, miR-504 expression level elevated less than 3.5-fold after radiotherapy) at different days after completing radiotherapy (Figure 3D). As expected, patients expressing high levels of miR-504 had a significantly (*p* = 0.013) greater percentage of tumor remaining compared to patients expressing lower levels of miR-504. Additionally, we examined the therapeutic effect of radiotherapy after 3 months in patients expressing high and low levels of miR-504 (Figure 3E). Patients were classified as either complete response (CR: 9/20 cases) or partial response (PR: 11/20 cases) groups. Results indicated that most patients expressing high levels of miR-504 experienced a lower therapeutic effect ratio of CR, but a higher ratio of PR compared to patients expressing low levels of miR-504, which further confirmed that higher miR-504 expression is associated with tumor radio-resistance and poor radio-therapeutic effect.

### Mitochondrial metabolic features and respiratory chain functions are altered in NPC radio-resistant and miR-504 over-expressing cells

**Figure 4:**
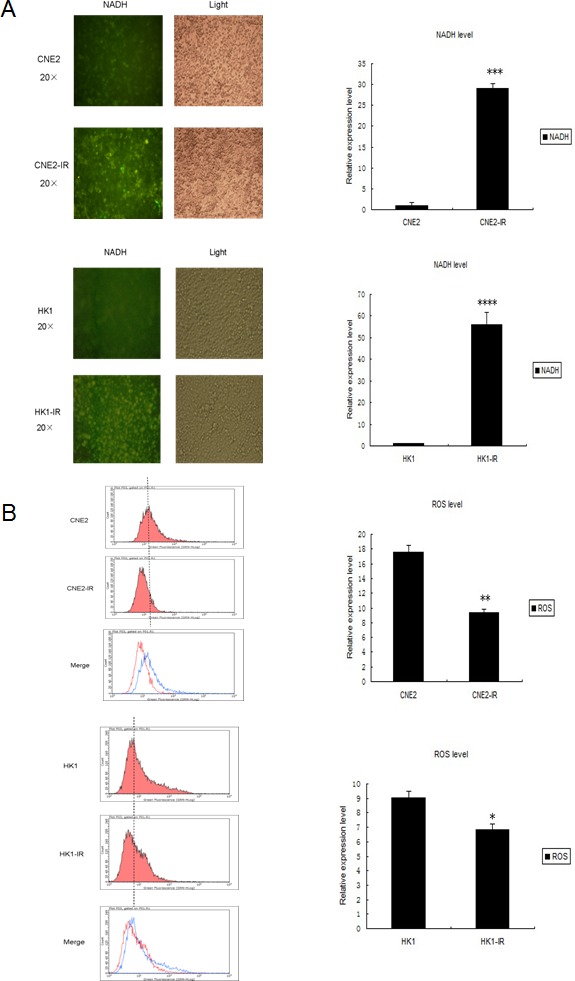

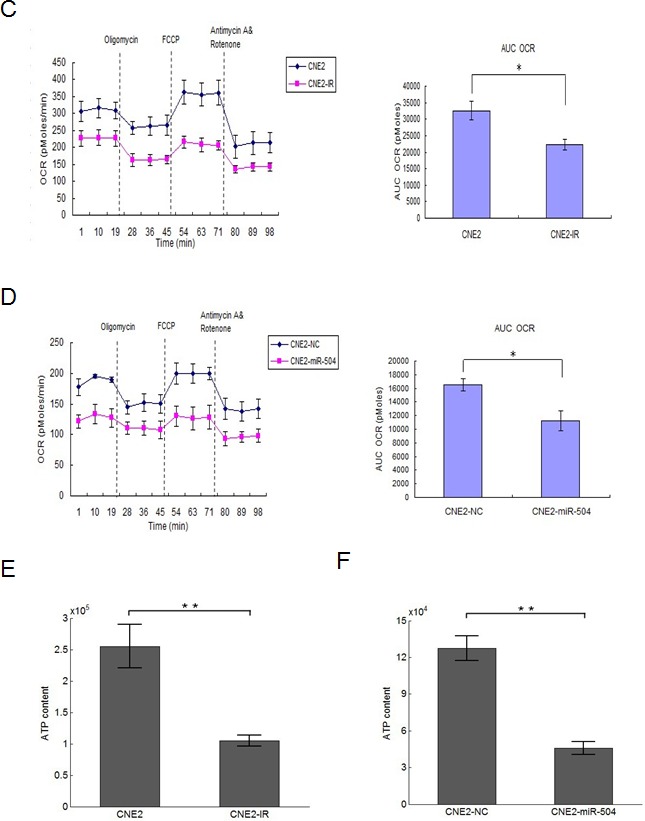
Changes in features of mitochondrial metabolism and respiratory chain functions in NPC radio-resistant cell lines and a miR-504 over-expressing cell line **A.** NADH levels in CNE2, CNE2-IR, HK1, and HK1-IR cells. Representative fluorescence images were shown on the left. The intensity of fluorescence was quantitated using Image J. Data on the right are expressed as mean values ± S.D. of three experiments. The asterisks (***, ****) indicate a significantly higher level of NADH in radio-resistant cells (*p* < 0.001, *p* < 0.0001, respectively). **B.** Reactive oxygen species (ROS) levels in CNE2, CNE2-IR, HK1, and HK1-IR cell lines. Representative images by flow cytometry were shown on the left. Data on the right are shown as mean values ± S.D. of three experiments. The asterisk(s) (*, **) indicate significantly less ROS level in radio-resistant cells (*p* < 0.05, *p* < 0.01, respectively). **C.** The oxygen consumption rate (OCR) and Area Under Concentration-Time Curve of OCR (AUC OCR) of CNE2 and CNE2-IR cells measured by the Seahorse XF-24 extracellular flux analyzer after the addition of four separate inhibitors at different time points. Data on the right are shown as mean values ± S.D. of three experiments. The asterisk (*) indicates a significant (*p* < 0.05) decreased AUC OCR value in radio-resistant cells. **D.** The oxygen consumption rate (OCR) and AUC OCR of CNE2-NC and CNE2-miR-504 measured by the Seahorse XF-24 extracellular flux analyzer after the addition of four separate inhibitors at different time points. Data on the right are shown as mean values ± S.D. of three experiments. The asterisk (*) indicates a significant (*p* < 0.05) decreased AUC OCR value in CNE2-miR-504 cells. **E.** The intracellular ATP content of CNE2 and CNE2-IR cells. **F.** The intracellular ATP content of CNE2-NC and CNE2-miR-504 cells. Data in **E.** and **F.** are shown as means ± S.D. of three experiments. The asterisks (**) indicate significantly less (*p* < 0.01) ATP content in CNE2-IR and CNE2-miR-504 cells compared to their respective controls.

During the radiation process, the function and metabolism of mitochondria can be disrupted and especially the balance between oxidative and reductive substances, including ROS and NADH. In addition, excessive amount of ROS can initiate apoptosis and therefore affect tumor radio-sensitivity [22, 23]. Thus, we next examined changes in ROS and NADH levels in NPC radio-resistant cell lines and miR-504 over-expressing cells. By transfecting the *Frex* plasmid, we were able to determine the relative level of NADH in NPC cells, their radio-resistant cells and miR-504 over-expressing cells. At 48 h after transfection, we observed NADH as the intensity of yellow fluorescence by microscope (Figure 4A and Figure S3A). Results indicated that the intensity of yellow fluorescence was about 30- to 55-fold higher in NPC radio-resistant cell lines and 47-fold higher in miR-504 over-expressing cell lines. This indicated that the level of NADH was significantly up-regulated in NPC radio-resistant CNE2-IR (*p* < 0.001), HK1-IR (*p* < 0.0001) cell lines and miR-504 over-expressing cell lines (*p* < 0.001) compared to parental cell lines. Additionally, we assessed the ROS level in these cells by staining them with the fluorescent probe, CM-H2DCF-DA, and measuring the intensity of green fluorescence by flow cytometry (Figure 4B and Figure S3B). The results demonstrated that ROS levels were much lower in CNE2-IR (*p* < 0.01), HK1-IR (*p* < 0.05) and miR-504 over-expressing cells (*p* < 0.05) compared to parental cells. These results suggested that mitochondrial metabolism was significantly altered in NPC radio-resistant cell lines and miR-504 over-expressing cell lines, as evidenced by high NADH but low ROS levels.

To further examine potential mitochondrial oxidative stress and respiratory chain dysfunction, we used the Seahorse XF-24 extracellular flux analyzer to determine the mitochondrial respiratory capacity and oxygen consumption rate (OCR) in CNE2 and CNE2-IR cell lines, after the addition of 4 separate inhibitors at different time points (Figure 4C). The data demonstrated that CNE2-IR cells exhibited a relatively lower OCR and a 1.4-fold less total oxygen consumption rate (AUC OCR: Area Under Concentration-Time Curve of OCR) compared to CNE2 cells (*p* < 0.05). The intracellular ATP content of these two cell types was also measured by ATPlite assay (Figure 4E) and data showed that CNE2-IR cells had 2.5-fold less intracellular ATP compared to CNE2 cells (*p* < 0.01). Similarly, we used the Seahorse XF-24 extracellular flux analyzer to measure the mitochondrial respiratory capacity and OCR in CNE2-NC and CNE2-miR-504 cell lines as before (Figure 4D). The data showed that CNE2-miR-504 cells exhibited a relatively lower OCR and 1.45-fold less total oxygen consumption rate (AUC OCR) than CNE2-NC cells (*p* < 0.05). The intracellular ATP content of each of these cell lines was measured by ATPlite assay (Figure 4F) and data showed that CNE2-miR-504 cells contained 2.9-fold less intracellular ATP than the CNE2-NC cells (*p* < 0.01). Moreover, we also measured ATP content in CNE2-IR-anti-NC and CNE2-IR-anti-miR-504 cells by ATPlite assay (Figure S4). These data suggested that the mitochondrial respiratory chain functions were hindered by miR-504 as evidenced by significant miR-504-associated decreases in oxygen consumption rate and ATP generation.

### miR-504 directly targets NRF1 and down-regulates the expression of the TFAM and OXPHOS complexes

**Figure 5:**
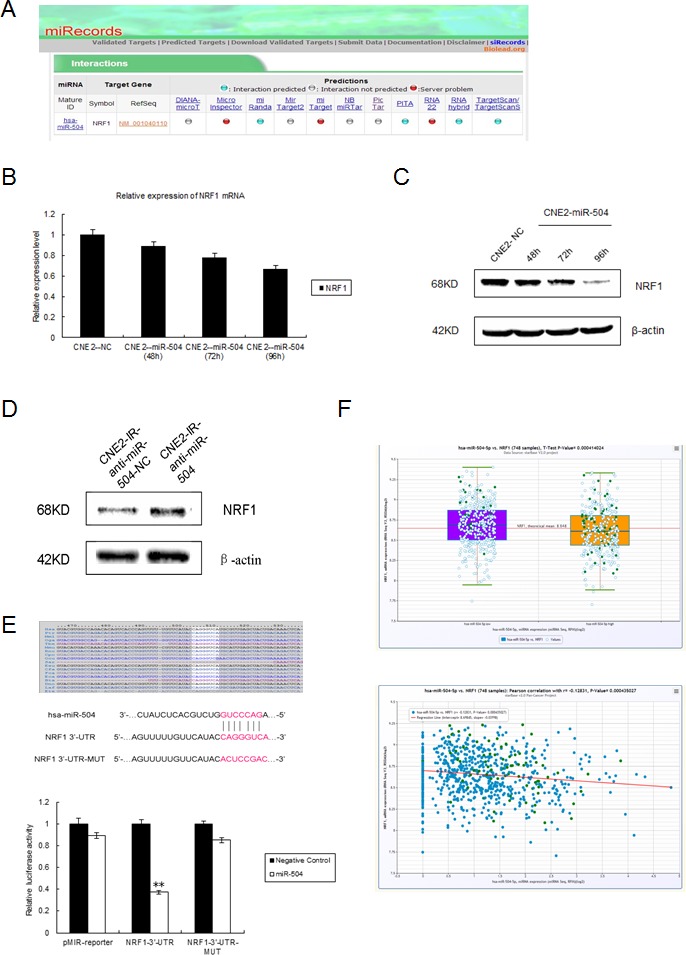

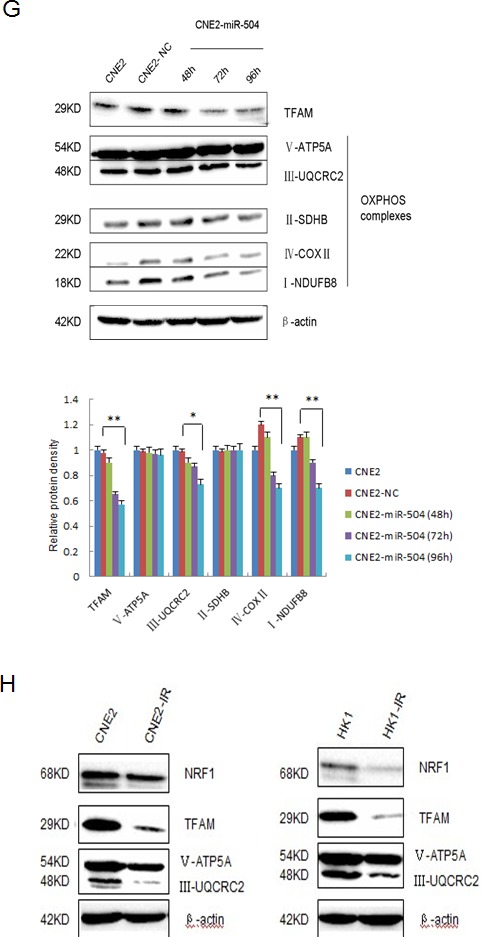
miR-504 directly targets NRF1 and down-regulates the expression of the TFAM and OXPHOS complexes **A.** The interaction between miR-504 and NRF1 can be predicted in several databases shown by miRecords. **B.** Relative expression level of NRF1 mRNA in the CNE2 cell line at 48, 72, or 96 h after transfection of a miR-504 precursor (CNE2-miR-504) or its negative control (CNE2-NC). Data are shown as mean values ± S.D. of three experiments. There is no statistical significance among these groups. **C.** The protein expression level of NRF1 in the CNE2 cell line at 48, 72, or 96 h after transfection of a miR-504 precursor (CNE2-miR-504) or its negative control (CNE2-NC). **D.** The protein expression level of NRF1 in the CNE2-IR cell line at 48 h after transfection of an inhibitor of miR-504 (CNE2-IR-anti-miR-504) or its negative control (CNE2-IR-anti-NC). **E.** The putative miR-504 binding site and its mutant site in the 3′-UTR of NRF1 are shown (upper panel). The relative luciferase activities of cells expressing a pMIR-reporter plasmid, NRF1-3′-UTR plasmid, or NRF1-3′-UTR-MUT plasmid when co-transfected with a miR-504 precursor or its negative control are shown (lower panel). Data are shown as mean values ± S.D. of three experiments. The asterisks (**) indicate a significant difference *(p* < 0.01). **F.** Clinical data showing the relative expression levels of miR-504, NRF1, and their correlation in 748 breast cancer samples were obtained from the starBase database. **G.** The expression levels of the TFAM and OXPHOS complexes in the CNE2 cell line at 48, 72, or 96 h after transfection of a miR-504 precursor and its negative control (NC) (upper panel). Relative protein band density was analyzed using Image J (lower panel). Data are shown as mean values ± S.D. of three experiments. The asterisk(s) (*, **) indicate a significant decrease in protein expression (*p* < 0.05, *p* < 0.01, respectively). **H.** The expression levels of factors related to mitochondrial metabolism, including NRF1, TFAM and OXPHOS complexes III and V, in CNE2, CNE2-IR, HK1, and HK1-IR cell lines.

Because miR-504 expression can lead to mitochondrial dysfunction, we used six different bioinformatics databases to predict its putative downstream target genes that are related to the regulation of mitochondrial function. Based on the results obtained from two integrated databases (starBase and miRecords), we divided the functions of miR-504 target genes into various categories (Figure S5A). No mitochondrial genes or their related pathway genes were discovered directly downstream of miR-504. However, miR-504 can target many transcription factors, including NRF1. NRF1 can act as a transcription factor to activate the expression of TFAM, which is essential for the transcription of all mitochondrial-encoded genes (Figure S5B). Meanwhile, the interactive relationships between miR-504 and NRF1 could be predicted simultaneously in four databases, including miRanda, PITA, RNAhybrid, and TargetScan (Figure 5A). Thus our next step was to validate NRF1 as a potential target of miR-504. First, we transiently transfected a miR-504 precursor and its negative control (NC) into CNE2 cells as described earlier. We extracted total RNA and the CNE2-NC and CNE2-miR-504 proteins at different time points (48, 72, 96 h) and used RT-PCR and Western blot to determine the mRNA and protein level, respectively, of NRF1 (Figure 5B, 5C). The data revealed that the mRNA expression level of *NRF1* was only slightly down-regulated in CNE2-miR-504 cells at different time points. However, the protein expression level of NRF1 was substantially down-regulated in CNE2-miR-504 cells, especially at the 96 h time point. We also transiently transfected the anti-miR-504 inhibitor and its anti-NC into CNE2-IR cells and at 48 h after transfection, we examined the protein level of NRF1 (Figure 5D). Results indicated that the protein expression level of NRF1 was obviously up-regulated in CNE2-IR-anti-miR-504 cells, suggesting that inhibiting the expression of miR-504 can lead to up-regulation of the NRF1 protein level.

From the TargetScan database, we found that NRF1 has one miR-504 binding site at 506-513 of the 3′-UTR, a position that is shared by many species, including humans (Figure 5E). We then constructed a NRF1-3′-UTR plasmid that contains the putative miR-504 binding site and a NRF1-3′-UTR-MUT plasmid that has mutant sequences in the miR-504 binding site. We co-transfected each plasmid with a miR-504 precursor or its NC into HEK 293 cell and conducted dual luciferase reporter assays (Figure 5E). The data indicated that the relative luciferase activity of NRF1-3′-UTR cells was lower than the pMIR-reporter-transfected cells and the NRF1-3′-UTR-MUT cells (*p* < 0.01), which suggested that miR-504 could directly bind to the 3′-UTR of NRF1 and inhibit its activity. Furthermore, we used the clinical resources in the starBase database [24, 25] and the Student's t-test and Pearson's correlation to analyze the expression levels and detect correlations between miR-504 and NRF1 in 748 breast cancer samples (Figure 5F). The results showed that the expression level of NRF1 was negatively correlated with the expression level of miR-504 (*p* < 0.001). Collectively, these data demonstrated that *NRF1* is a target gene of miR-504.

Next, we over-expressed miR-504 in CNE2 cells and harvested them at different time points (48, 72, 96 h) to examine the expression levels of two mitochondrial metabolism-related factors, the TFAM and OXPHOS complexes, which are downstream of NRF1 (Figure 5G). Results indicated that the protein levels of the TFAM and OXPHOS complexes I, III, and IV were significantly down-regulated by miR-504 over time and especially at the 96 h time point. Additionally, we determined the expression levels of these mitochondrial metabolism-related factors in two NPC radio-resistant cell lines (Figure 5H). Data indicated that except OXPHOS complex V, the other three mitochondrial metabolism-related factors NRF1, TFAM and OXPHOS complex III were obviously down-regulated in radio-resistant cell lines, which is consistent with the miR-504 over-expression effects.

### Down-regulation of NRF1 contributes to NPC radio-resistance and NRF1 mediates the function of miR-504 in determining NPC radio-resistance

**Figure 6:**
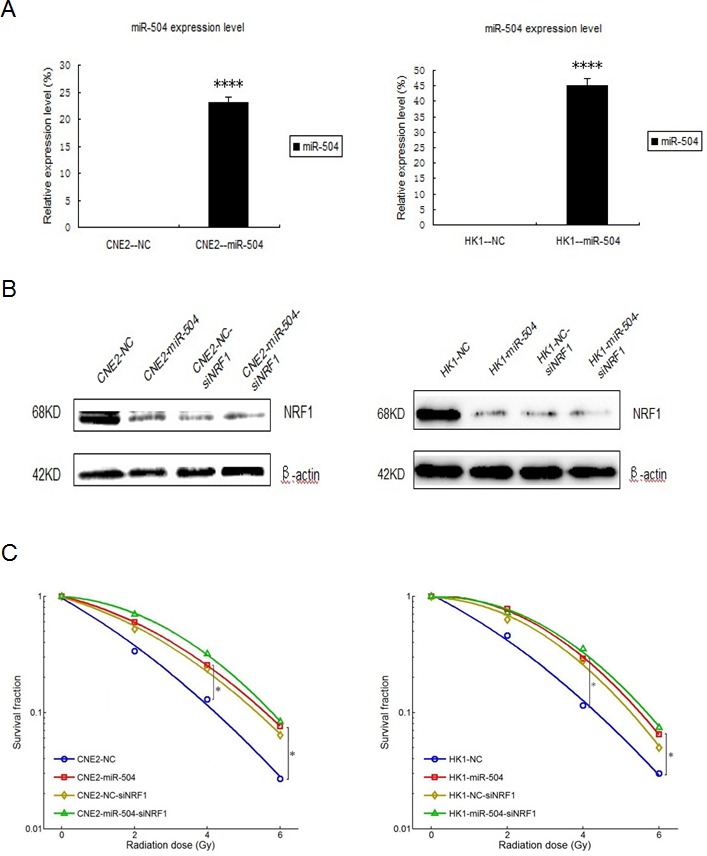

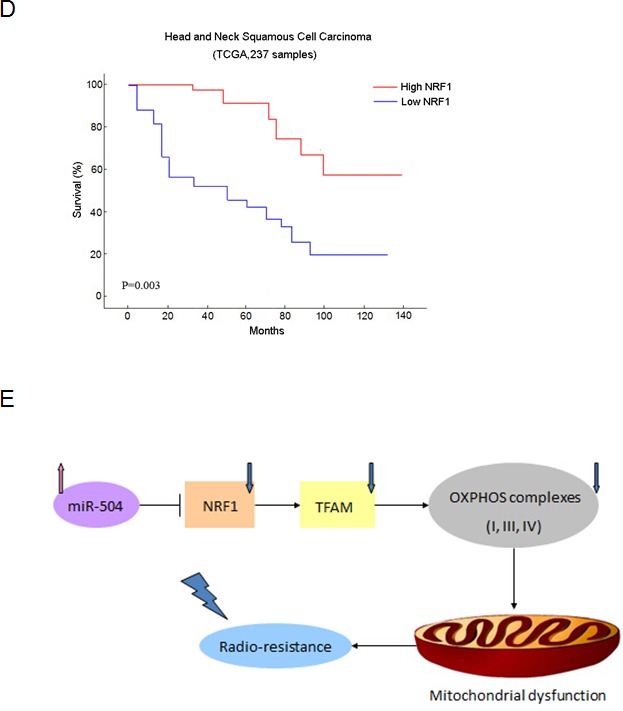
Down-regulation of NRF1 contributes to NPC radio-resistance and NRF1 mediates the function of miR-504 in determining NPC radio-resistance **A.** The transfection efficiency of miR-504 after stable transfection of lentiviral vectors of miR-504 and its negative control (NC) into CNE2 and HK1 cells. The asterisks (****) indicate a significant increase in miR-504 (*p* < 0.0001). **B.** The expression level of NRF1 in these cells after transfection with siNRF1. **C.** The survival curves for CNE2-NC, CNE2-miR-504, HK1-NC, HK1-miR-504 and their siNRF1 cell lines. The asterisk (*) indicates a significant decrease in survival (*p* < 0.05). **D.** The survival curve for head and neck squamous cell carcinoma patients corresponds to the different expression level of NRF1. Data were obtained from The Cancer Genome Atlas (TCGA) database. **E.** A schematic model based on our findings.

To further explore the mechanisms of miR-504 in NPC radio-resistance, we stably transfected the lentiviral vectors of miR-504 and its negative control (NC) into CNE2 and HK1 cells to establish two miR-504 stably over-expressing cell lines, CNE2-miR-504 and HK1-miR-504. Then, we determined the expression levels of miR-504 in these two cell lines (Figure 6A). The data showed that the transfection efficiency of miR-504 lentiviral vectors was high and the miR-504 expression levels were significantly up-regulated in CNE2-miR-504 (*p* < 0.0001) and HK1-miR-504 (*p* < 0.0001) cells. Next, we used siRNA strategy to knock down the expression of NRF1 in these cells and determine the protein expression level of NRF1 (Figure 6B). Results indicated that the protein level of NRF1 can be effectively down-regulated by siNRF1 in these two cell types. Moreover, we treated each cell type with different doses of IR (0, 2, 4, or 6 Gy) and performed the colony formation assay (Figure 6C). The data showed that over-expression of miR-504 or knock-down of NRF1 could lead to high SF in NPC cells, which also suggests that the up-regulation of miR-504 or down-regulation of NRF1 can contribute to NPC radio-resistance. Moreover, clear SF differences were observed between CNE2-NC and CNE2-miR-504 cells or HK1-NC and HK1-miR-504 cells. However, the differences were reduced between CNE2-NC-siNRF1 and CNE2-miR-504-siNRF1 cells or HK1-NC-siNRF1 and HK1-miR-504-siNRF1 cells, which suggests that the function of miR-504 in determining NPC radio-resistance is mediated by NRF1. Furthermore, we used The Cancer Genome Atlas (TCGA) database to examine the overall survival rate of head and neck squamous cell carcinoma due to different expression levels of NRF1 (Figure 6D). The survival curve demonstrated that a lower expression level of NRF1 leads to poor survival rate in cancer patients, which is at least partly due to the enhancement of radio-resistance characteristics in patients during radiotherapy.

## DISCUSSION

Multiple miRNAs, including miR-141, miR-29c, miR-26a, miR-218, miR-200a, and miR-18a, have been reported to play key roles in tumor growth, proliferation, progression and metastasis of NPC [26-31]. Additionally, the occurrence of specific miRNA expression signatures in blood serum and plasma of human cancers has been recently observed, suggesting that specific types of circulating miRNAs can act as promising novel non-invasive biomarkers for early onset or relapse of a wide range of solid cancers. In NPC, miR-17, miR-20a, miR-29c, and miR-223 have been reported to be essential serum biomarkers [32]. However, the function and clinical application of miR-504 in NPC is relatively poorly studied. Here, our results showed that in serum samples from NPC patients, miR-504 was obviously up-regulated during different weeks of radiotherapy and correlated with TNM stage and total tumor volume. An evaluation of the radio-therapeutic effect in patients at three months after radiotherapy revealed that patients expressing high levels of miR-504 exhibited a relatively lower therapeutic effect ratio of CR, but a higher ratio of PR compared to patients expressing low miR-504 levels. These findings might provide new evidence supporting the use of miR-504 as a non-invasive radio-resistant biomarker to predict tumor radiation response at the early stages and also aid in monitoring radio-therapeutic effects and prognosis after radiotherapy.

Mitochondria are essential for performing diverse cellular functions and disruption of their normal metabolism is a common feature in radio-resistant tumor cells. By disturbing the function of apoptosis-related factors, such as Bax, Bcl-2, Bcl-xL, Mcl-1 and p53, in mitochondria, the radio-resistant cells can evade apoptotic-related death induced by radiation treatment [33-35]. Additionally, in radio-resistant cells, the levels of mitochondrial oxidants and antioxidants, including ROS, H_2_O_2_, O_2_^−^, SOD, NADH, GSH, and the redox potential of mitochondrial membranes, are markedly altered, resulting in an imbalance of the oxidative redox system and initiation of ROS-induced anti-apoptotic processes [36, 37]. Moreover, in the mitochondria of radio-resistant cells, the tricarboxylic acid (TCA) cycle and OXPHOS complexes [38, 39] are at least partially impaired. All of these disruptive factors can contribute to a cell's ability to gain a radio-resistant phenotype.

NRF1 is a nuclear gene encoded transcription factor, which has been shown to bind the antioxidant response element (ARE) and regulate the expression of a number of genes involved in oxidative stress [40]. Examples of these types of genes include *heme oxygenase*, *glutathione peroxidase, heavy and light chains of ferritin*, and *superoxide dismutase*, which each functions to protect cells from ROS-mediated injury [41]. TFAM, which was cloned as a transcription factor for mtDNA and is under the regulation of NRF1, is an essential protein that binds mtDNA with or without sequence specificity to regulate both mitochondrial transcription initiation and mtDNA copy number [42]. TFAM has also been shown to be essential for the maintenance of mtDNA integrity and is important for maintaining proper cellular functions under both physiological and pathological conditions [43]. In our study, we identified NRF1 as a direct target of miR-504 that is involved in the regulation of the tumor radiation response. These findings have helped build a novel connection between mitochondrial dysfunction, especially abnormal OXPHOS processes, and tumor radio-resistance mediated by miRNA regulatory mechanisms.

The mitochondrial OXPHOS system is mainly responsible for the production of energy in the cell. The process occurs in the inner mitochondrial membrane and is mediated by the five enzymatic complexes of the mitochondrial respiratory chain [44]. The subunits constituting these complexes have a dual genetic origin, mitochondrial or nuclear. Although many nuclear DNA mutations involve genes coding for subunits of the respiratory complexes, the majority of mutations found, to date, affect factors that do not form part of the final complexes. These assembly factors or chaperones have multiple functions ranging from cofactor insertion to proper assembly/stability of the complexes [45, 46]. In our study, the data showed that not all of the OXPHOS complexes, including OXPHOS complexes II and V, can be down-regulated by TFAM in NPC radio-resistant cells. This suggests that the expression or the assembly of these two complexes can be modulated by nuclear genes or are under the control of both mtDNA and nuclear genes. Overall, our results confirm that mitochondria become dysfunctional and metabolism is disrupted in radio-resistant and miR-504 over-expressing cells and also suggest that miR-504 has a close relationship with the radio-resistant phenotype.

In summary, our findings indicated that miR-504 is up-regulated in NPC radio-resistant cells and miR-504 could directly down-regulate the expression of NRF1 and lead to radio-resistance in NPC cells. By disturbing mitochondrial-mediated oxidative responses, miR-504 allows NPC cells to gain radio-resistant characteristics (Figure 6E). However, more detailed mechanisms and pathways regarding the way in which mitochondrial dysfunction influences apoptosis of tumor cells and contributes to tumor radio-resistance need to be explored. In addition, the potential of miR-504 and NRF1 as molecular markers and therapeutic targets for the treatment of NPC needs to be further investigated.

## MATERIALS AND METHODS

### Establishing NPC radio-resistant cell lines

We used two NPC radio-resistant cell lines, CNE2-IR and HK1-IR, in our study. The CNE2-IR cell line was established as previous described [19, 20] and the HK1-IR cell line was established by exposing HK1 cells in exponential growth phase to a repeated IR dose of 4 Gy each. An interval of 3 to 8 weeks between each IR dose allowed the surviving cells to regenerate. The whole process of IR and culture lasted for about 1 year with the total IR dose at 80 Gy. We refer to the surviving cell line as HK1-IR.

### Cell culture

Human NPC cell lines, including CNE2 and HK1, and their radio-resistant cell lines CNE2-IR and HK1-IR were cultured in RPMI 1640 with 10% fetal bovine serum (FBS), 1% glutamine, and 1% antibiotics. All cell lines were grown in a humidified incubator at 37°C with 5% CO_2_.

### Cell line short tandem repeat (STR) genotyping

CNE2 and HK1 cells and their radio-resistant companion cell lines, CNE2-IR and HK1-IR cells, were collected for genomic DNA (gDNA) extraction, which was followed by STR genotyping. The whole process was performed by Microread, Beijing, P.R.China.

### Patients and serum samples

Patient sample collection was performed according to the protocols approved by the Human Ethics Committee of Xiangya Hospital, Central South University, Changsha, China. Following ethical approval and written informed consent, serum samples were collected from 20 NPC patients at Xiangya Hospital. For each patient, the serum samples were collected at four different weeks (0, 2, 4, 6 weeks) during radiotherapy with a total number of 80 NPC patients' serum samples. All the patients had been diagnosed with NPC pathologically and their relevant demographic and clinical pathological details were obtained.

### Evaluation criteria of radio-therapeutic effects

All tumor volumes were imaged from NPC patients and measured by Magnetic Resonance Imaging (MRI) by two different specialized radiologists. Three months after radiotherapy, the therapeutic effect was evaluated by two parameters–complete response (CR) and partial response (PR). CR indicates that all tumor lesions had disappeared whereas PR indicates that the tumor lesions shrunk to no less than 30% of the original total tumor volume.

### Serum miRNA and RNA extraction

For serum collection, venous blood (5 ml) was collected in the morning before breakfast from NPC patients before any therapeutic procedures and then extracted using a mirVana PARIS kit (AM1556, Ambion, Austin, TX, USA) following the manufacturer's protocol for liquid samples. Cellular miRNA extraction was performed using mirVana^TM^ miRNA Isolation Kit (AM1560, Ambion) following the manufacturer's instructions. Total RNA extraction was performed using TRIzol Reagent (Invitrogen, Carlsbad, CA, USA) following the manufacturer's instructions.

### Real-time PCR assays

The miR-504 quantitative real-time PCR assay was performed using TaqMan^®^ MicroRNA Assays (ID: 002084, Applied Biosystems, Carlsbad, CA, USA); and U6 snRNA (ID: 001973, Applied Biosystems, Carlsbad, CA, USA) was used as an internal control. For the serum miR-504 quantitative real-time PCR assay, miR-24 (ID: 000402, Applied Biosystems) was used as an internal control. The relative expression level was determined as 2^−ΔΔCt^.

For the mRNA quantitative real-time PCR (RT-PCR) assay, NRF1 expression was detected using SYBR Green I chemistry (Power SYBR Green PCR Master Mix, ABI Inc., Carlsbad, CA, USA). Real-time PCR and data collection were performed with an ABI 7500 sequence detection system. The housekeeping gene, *β-actin*, was used as an internal control. The relative fold changes were calculated using the 2^−ΔΔCt^ method. The primers were as follows: *NRF1*: forward: 5′-CCACGTTACAGGGAGGTGAG-3′ and reverse: 5′-TGTAGCTCCCTGCTGCATCT-3′); *β-actin*: (forward: 5′-TTCCAGCCTTCCTTCCTGGG-3′ and reverse: 5′-TTGCGCTCAGGAGGAGCAAT-3′).

### Western blot analysis

The antibodies for Western blot were as follows: NRF1 (sc-101102, Santa Cruz, CA, USA), TFAM (OAAB00276, Aviva System Biology, San Diego, CA, USA), MitoProfile^®^ Total OXPHOS Human WB Antibody Cocktail (ab110411, Abcam) and β-actin (sc-8432, Santa Cruz).

### Transient transfection

Cells were transfected with SMARTpool SiGENOME *NRF1* siRNA (M-017924-01-0005, Thermo Scientific, Carlsbad, CA, USA), miR-504 Pre-miR miRNA Precursor Molecules (Cat#: PM12429, Ambion), or Pre-miR miRNA Precursor Molecules Negative Control #1 (Cat#: AM17110, Ambion), and anti-miR™ miR-504 Inhibitor (Cat#: AM29742, Ambion), or anti-miR™ miRNA Inhibitor Negative Control #1 (Cat#: AM17010, Ambion) using Lipofectamine 2000 (Invitrogen) according to the manufacturer's instructions.

### Lentivirus transfection

Human hsa-miR-504 and its control lentiviral vector were constructed by GeneChem, Shanghai, P.R.China. According to the manufacturer's instructions (Lentiviral Vector Particle, 5.0, GeneChem), they were respectively transfected into CNE2 and HK1 cells.

### Analysis of predicted miRNAs targets

The putative targets of miRNAs were analyzed using the following six respective databases: miRecords (http://mirecords.biolead.org/), miRanda (http://www.microrna.org//miranda.html), TargetScan (http://genes.mit.edu/targetscan), PITA (http://genie.weizmann.ac.il/pubs/mir07/mir07_data.html), RNAhybrid (http://bibiserv.techfak.uni-bielefeld.de/rnahybrid/), and starBase (http://starbase.sysu.edu.cn/).

### Luciferase reporter assays

3′-UTR sequences and the mutant sequences of NRF1 containing the putative miR-504 target sites were cloned in the plasmid SV40-Luc-MCS-pMIR-report-Vector (GeneChem, Shanghai, P.R.China). Luciferase reporter assays were performed in HEK293 cells using the Dual Luciferase Reporter Assay (Cat#: E1910, Promega, Madison, WI, USA). Firefly luciferase values were normalized to *Renilla,* and the ratio of firefly/*Renilla* activity is presented.

### Apoptosis assay

Cells were stained by Annexin V-PE and 7-AAD using the Annexin V-PE/7-AAD apoptosis kit (KeyGEN Biotech, Nanjing, Jiangsu, P.R. China) according to the manufacturer's instructions. Storing and processing of data were accomplished using FACScan software.

### MTS assay

Cells were seeded into 96-well plates and incubated for 0, 24, 48, or 72 h. The MTS reagent was added to each well and cell viability was assessed by MTS assay according to the instructions provided (Promega).

### BrdU staining assay

10^5^ to 10^8^ dividing cells were labeled with 10 μM BrdU for 45 min at 37°C, then fixed and stained with 5 μl of Anti-BrdU fluorochrome conjugated antibody for 20-30 min at room temperature in the dark, and followed by flow cytometry to test the green fluorescence intensity. All the manufacturer's instructions were referred to BrdU Staining Kit for Flow Cytometry FITC (Cat#: 8811-6600, eBioscience).

### Seahorse XF-24 metabolic flux analysis

Oxygen consumption rate (OCR) was measured using a Seahorse XF-24 extracellular flux analyzer (Seahorse Bioscience, Chicopee, MA, USA). Twenty four hours before the experiment, CNE2, CNE2-IR, CNE2-NC, and CNE2-miR-504 cells were cultured on Seahorse XF-24 plates at a density of 2 × 10^4^ cells per well. On the day of metabolic flux analysis, the culture medium was replaced with 600 μl of unbuffered DMEM and incubated at 37°C in a non-CO_2_ incubator for 1 hour. Then the following mitochondrial inhibitors were sequentially injected: oligomycin (1 μM), carbonycyanide p-(trifluoromethoxy) phenylhydrazone (FCCP) (0.3 μM), antimycin A and rotenone (1 μM). OCR was automatically calculated by the Seahorse XF-24 software.

### Determination of cellular ROS levels

Cellular ROS levels were measured by staining cells with CM-H2DCF-DA (C6827, Invitrogen) for 60 min, followed by flow cytometry, and data were analyzed using CellQuest Pro software.

### Determination of cellular NADH level

CNE2 and HK1 cells and their respective radio-resistant cell lines, CNE2-IR and HK1-IR, were transfected with the *Frex* plasmid (kindly provided by Prof. Yang Yi, East China University of Science and Technology, P.R.China) [21]. At 48 h after transfection, the intensity of yellow fluorescence was observed under a fluorescence microscope and quantitated using Image J software, which represented the cellular NADH level.

### Measurement of intracellular ATP content

Intracellular ATP content was measured using the Luminescence ATP Detection Assay System (ATPlite Perkin Elmer Life Sciences, Waltham, MA, USA). The ATPlite assay is based on the production of light generated by the reaction of ATP with added luciferase and D-luciferin (similar to firefly luciferase assay). The emitted light is proportional to ATP concentration. In brief, 8 ×10^3^ cells per well were first plated in the 96-well culture plate for 24 h and then 50 μl of mammalian cell lysis buffer was added to each well and plates shaken for 5 min. Then 50 μl of substrate solution were added to each well and the plate was shaken at 700 rpm for another 5 min. The plate was placed in the dark for 10 min and then the luminescence was measured.

### Colony formation assay

Cells were seeded into a six-well plate and then incubated for 24 h to allow settling. Cells were then treated with a range of ionizing radiation (IR) doses (0, 2, 4, 6 Gy) using a gamma irradiator. After 12-14 days, cells were washed with PBS, fixed in methanol and stained with crystal violet. The survival curves were drawn using the GraphPad Prism 5 software program (GraphPad Software, La Jolla, CA, USA).

### Statistical analysis

All quantitative data are expressed as mean values ± S.D. of at least 3 independent experiments. Statistical analysis was performed using SPSS17.0. Significant differences between two groups were compared using the Student's t-test, and comparisons among more than two groups were performed using analysis of variance (ANOVA). The correlation analysis was conducted using Pearson's correlation coefficient. A probability value of P < 0.05 was considered statistically significant.

## SUPPLEMENTARY MATERIAL TABLES AND FIGURES


