# Supplementary material for: miR-504 mediated down-regulation of nuclear respiratory factor 1 leads to radio-resistance in nasopharyngeal carcinoma
Source: Oncotarget. 2015 May 14;6(18):15995–6018. doi: 10.18632/oncotarget.4138 (PMC4599252; doi:10.18632/oncotarget.4138)
Supplement: Supplementary file 1 [file oncotarget-06-15995-s001.pdf]

miR-504 mediated down-regulation of nuclear respiratory factor 1 leads to radio-resistance in nasopharyngeal carcinoma

Supplementary Material

Table S1: The specific 20 gene loci of STR genotyping in NPC cell lines and their radio-resistant cell lines

STR genotyping of 20 gene loci in CNE2

CNE2（ID: XB1175）			
Marker	Allele 1	Allele 2	Allele 3
D19S433	13	13	
D5S818	11	12	
D21S11	30	30	
D18S51	13	16	
D6S1043	11	14	18
D3S1358	15	18	
D13S317	10	12	13.3
D7S820	10	12	
D16S539	9	10	
CSF1PO	10	11	
PentaD	9	12	
Amelogenin	X	X	
vWA	14	16	
D8S1179	12	16	
TPOX	8	12	
PentaE	17	20	
TH01	6	7	9
D12S391	20	21	

D2S1338	17	23	
FGA	18	21	

STR genotyping of 20 gene loci in CNE2-IR

CNE2-IR（ID: XB1176）		
Marker	Allele 1	Allele 2
D19S433	13	14
D5S818	11	12
D21S11	27	28
D18S51	16	16
D6S1043	18	19
D3S1358	15	18
D13S317	12	13.3
D7S820	8	12
D16S539	9	10
CSF1PO	9	10
PentaD	8	15
Amelogenin	X	X
vWA	16	18
D8S1179	12	13
TPOX	8	12
PentaE	7	17
TH01	7	7
D12S391	20	25
D2S1338	17	17
FGA	21	21

STR genotyping of 20 gene loci in HK1

HK1（ID: XB1173）		
Marker	Allele 1	Allele 2
D19S433	14	14
D5S818	11	13
D21S11	28	29
D18S51	17	17
D6S1043	12	20
D3S1358	15	15
D13S317	11	11
D7S820	8	11
D16S539	9	11
CSF1PO	11	12
PentaD	9	12
Amelogenin	X	X
vWA	18	19
D8S1179	11	13
TPOX	8	11
PentaE	15	18.4
TH01	7	9
D12S391	17	19
D2S1338	20	23
FGA	26	26

STR genotyping of 20 gene loci in HK1-IR

HK1-IR（ID: XB1174）		
Marker	Allele 1	Allele 2
D19S433	14	14
D5S818	11	13
D21S11	28	29
D18S51	17	17
D6S1043	12	20
D3S1358	15	15
D13S317	11	11
D7S820	8	11
D16S539	9	11
CSF1PO	11	11
PentaD	9	12
Amelogenin	X	X
vWA	18	19
D8S1179	11	13
TPOX	8	11
PentaE	15	18.4
TH01	7	9
D12S391	17	19
D2S1338	20	23
FGA	26	27

Table S2: Clinical features of NPC patients undergoing radiotherapy

Number	*Gender*	*Age*	*Primary tumor volume (cm^3)*	*Nodes metastasis volume (cm^3)*	*Total tumor volume (cm^3)*	*TNM stage*
1	Male	73	20.75	21.31	42.06	T2N3M0 IVa
2	Male	49	14.56	9.87	24.43	T2N2M0 III
3	Male	36	27.71	3.4	31.11	T2N1bM0 II
4	Female	32	9.49	30.69	40.18	T3N2M0 III
5	Male	42	21.54	4.84	26.38	T3N1aM0 III
6	Female	67	44.56	4.87	49.43	T3N1M0 III
7	Male	35	19.92	3.74	23.66	T2N1aM0 II
8	Female	48	34.68	13.14	47.82	T3N1M0 III
9	Male	29	22.43	32.5	54.93	T2N3M0 IVa
10	Male	50	15.74	38.36	54.1	T3N3M0 IVa
11	Male	44	46.75	27.79	74.54	T4N2M0 IVa
12	Female	50	16.98	28.9	45.88	T2N2M0 III
13	Female	43	42.9	10.69	53.59	T4N2M0 IVa
14	Male	50	29.63	36.82	66.45	T3N3M0 IVa
15	Male	60	12.43	47.87	60.3	T2N3M0 IVa
16	Female	45	11.94	1.86	13.8	T2N1bM0 II
17	Female	54	45.24	21.52	66.76	T4N3M0 IVa
18	Female	60	15.9	25.33	41.23	T3N1bM0 III
19	Female	46	5.59	0	5.59	T3N0M0 III
20	Female	66	12.22	6.32	18.54	T2N2M0 III

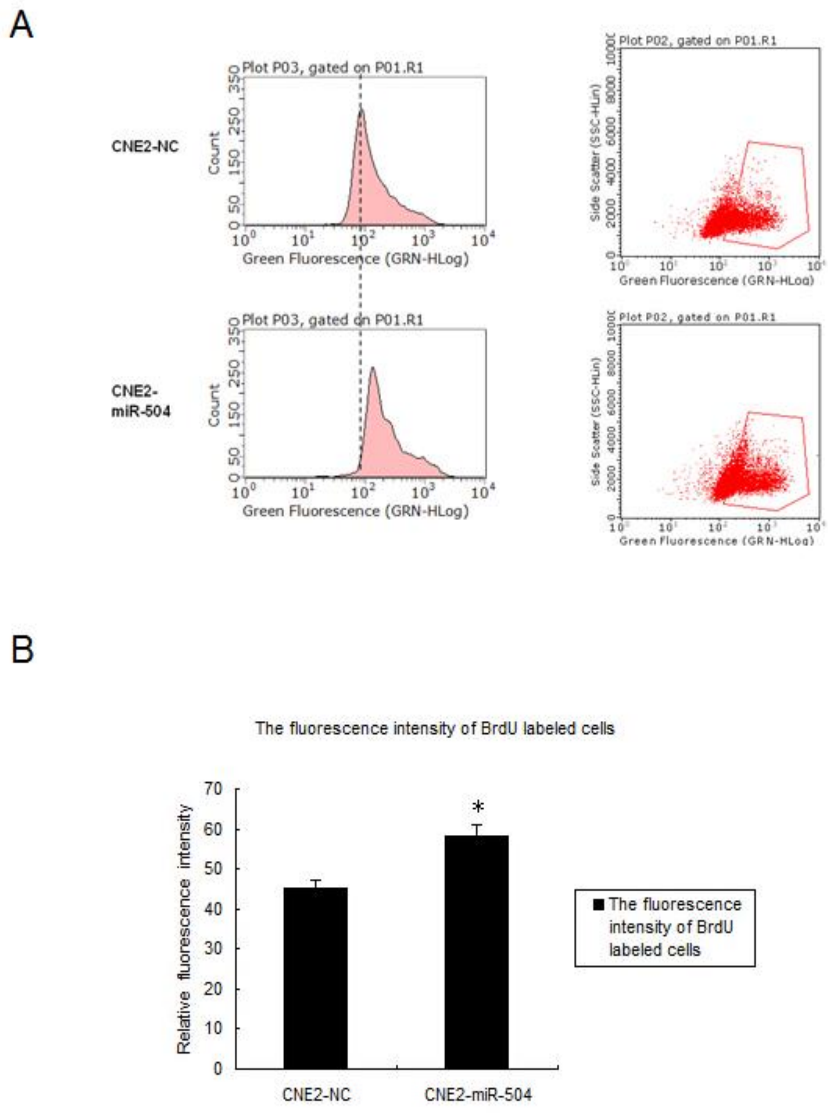

Figure S1: Using BrdU staining method to verify that miR-504 can promote NPC cells growth. (A) BrdU labeled positive cells and their green fluorescence intensity in CNE2-NC and CNE2-miR-504 cell lines. Representative images by flow cytometry were shown. (B) Calculation of the fluorescence intensity of BrdU labeled cells. Data are shown as mean values ± S.D. of three experiments. The asterisk (*) indicate significantly higher fluorescence intensity of BrdU labeled cells in miR-504 over-expressed group ($p < 0.05$).

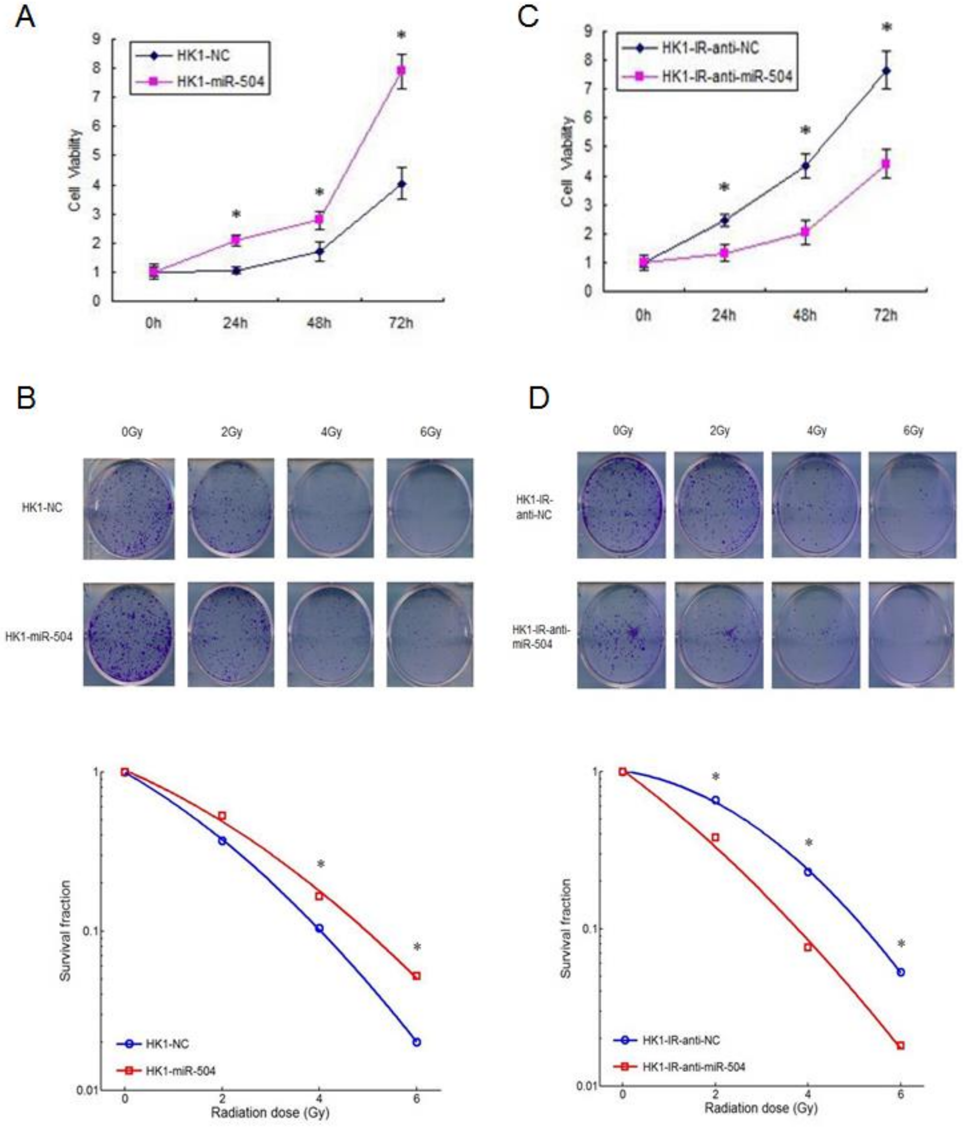

Figure S2: miR-504 promotes HK1 cell growth and leads to radio-resistance of the HK1 cell line. (A) Results of an MTS assay to assess viability of the HK1 cell line at 48 h after transfection of miR-504 precursor (HK1-miR-504) or its negative control (HK1-NC). (B) Results of a colony formation assay and survival curves for HK1-NC and HK1-miR-504 cells. The asterisk (*) indicates a significant difference ($p < 0.05$). (C) Results of an MTS assay to assess viability of the HK1-IR cell line at 48 h after transfection of an inhibitor of miR-504 (HK1-IR-anti-miR-504) or its negative control (HK1-IR-anti-NC). For A and C, data are shown as mean values ± S.D. of three experiments, and the asterisk (*)

indicates a significant difference ($p < 0.05$). (D) Results of a colony formation assay and survival curves for HK1-IR-anti-NC and HK1-IR-anti-miR-504 cells. The asterisk (*) indicates a significant difference ($p < 0.05$).

A

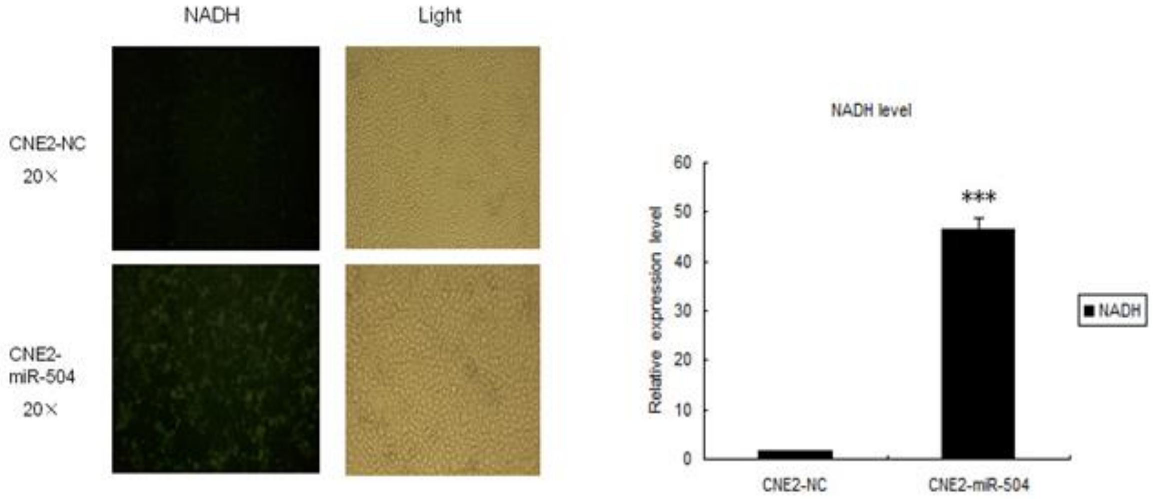
NADH
Light
CNE2-NC
20×
CNE2-
miR-504
20×
NADH level
60
50
40
30
20
10
0
Relative expression level
***
NADH
CNE2-NC
CNE2-miR-504

B

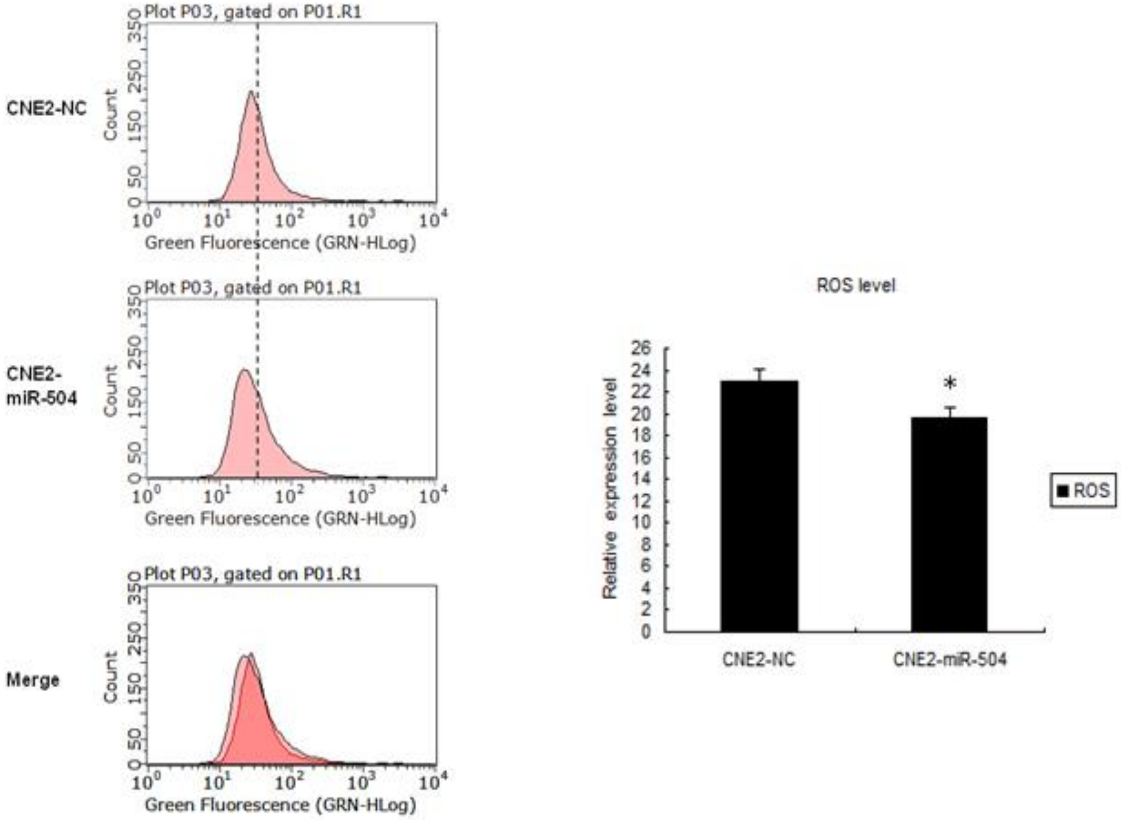
CNE2-NC
CNE2-
miR-504
Merge
Plot P03, gated on P01.R1
Count
0
50
150
250
350
10^0
10^1
10^2
10^3
10^4
Green Fluorescence (GRN-HLog)
ROS level
26
24
22
20
18
16
14
12
10
8
6
4
2
0
Relative expression level
*
ROS
CNE2-NC
CNE2-miR-504

Figure S3: Changes of the NADH and ROS levels in a miR-504 over-expressing cell line. (A) NADH levels in CNE2-NC and CNE2-miR-504 cell lines. Representative fluorescence images were shown on the left. The intensity of fluorescence was quantitated using Image J. Data on the right are expressed as mean values ± S.D. of three experiments. The asterisks (***) indicate a significantly higher level of NADH in miR-504 over-expressing cells ($p < 0.001$). (B) Reactive oxygen species (ROS) levels in CNE2-NC and CNE2-miR-504 cell lines. Representative images by flow cytometry were shown on the left. Data on the right are shown as mean values ± S.D. of three experiments. The asterisk (*) indicate significantly less ROS level in miR-504 over-expressing cells ($p < 0.05$).

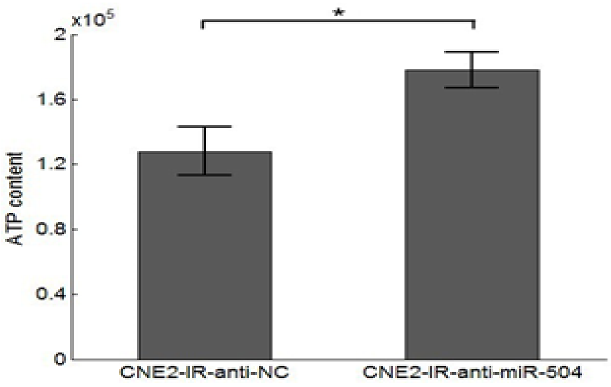

Figure S4: The intracellular ATP content of CNE2-IR cells transfected with an inhibitor of miR-504 (CNE2-IR-anti-miR-504) or negative control (CNE2-IR-anti-NC) as assessed using the ATPlite assay. The asterisk (*) indicates a significant difference ($p < 0.05$).

A

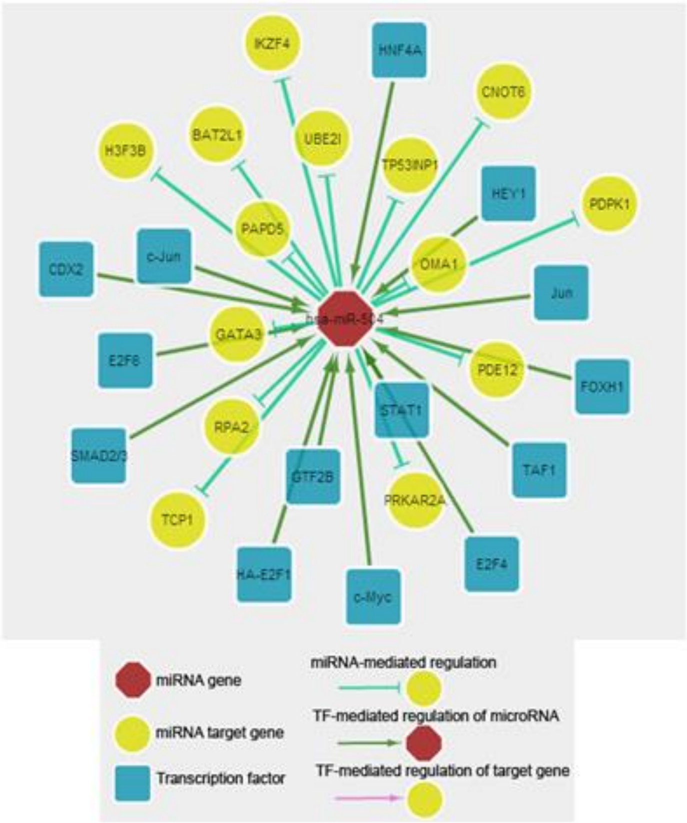

Analysis using ChIPBase database (http://deepbase.sysu.edu.cn/chipbase/index.php)

B

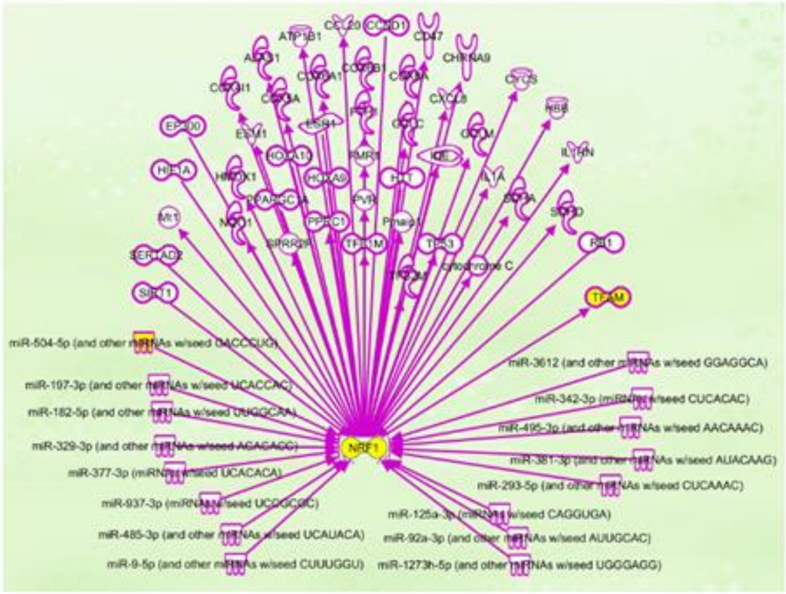

Analysis using Ingenuity Pathway Analysis (IPA) (http://www.ingenuity.com/products/ipa)

Figure S5: Using the bioinformatics tools to analysis the regulatory network of miR-504 and NRF1. (A) Using ChIPBase database to analysis the miR-504-centered regulatory network. (B) Using Ingenuity Pathway Analysis (IPA) software to analysis the NRF1-centered regulatory network.
